# Studying the Synthesis of Silver Nanocubes and Their Structural Evolution under Controlled Galvanic Reactions

**DOI:** 10.1021/acs.jpcc.5c03561

**Published:** 2025-07-25

**Authors:** Anika Guo, Nicolas Hall, Teagan Hamlett, John R. Crockett, Annabella Talbott, Tosin Ogunrinola, Ayomide Oluwafemi, Meghan Burke, Qian Chen, Ying Bao

**Affiliations:** 1 Department of Chemistry, 1632 Western Washington University, Bellingham, Washington 98225-9008, United States; 2 Department of Materials Science and Engineering, The Grainger College of Engineering, University of Illinois Urbana−Champaign, Urbana, Illinois 61801-3028, United States

## Abstract

Galvanic replacement reaction (GRR) with or without a reducing agent is the most commonly used strategy to transform Ag nanocubes (AgNCs) into nanocages, and under this approach, some limited control over the nanocages’ morphology and composition has been previously demonstrated. However, there is a lack of systematic study of GRR using other factors beyond a reducing agent to finely tailor the morphology and composition (mono- or bimetallic) of the nanocage formation. In addition, most previous work synthesizes AgNCs using the polyol process method, which has a number of drawbacks. In this work, we synthesized the AgNCs using the gold seed-mediated method and found that their morphology and yield were significantly impacted by both the ratio of silver ions to gold seed volume as well as the concentration of the CTAC surfactant. A detailed study was then carried out on transforming the synthesized AgNCs to nanocages under GRR while manipulating synthesis inputs, including the use of a reducing agent, adjusting the injection rate, and increasing the reaction temperature. Compared to a traditional GRR synthesis, manipulating these different inputs can result in a dramatically different structural evolution for the nanocages, which will impact their optical properties. This understanding allows for the morphology and composition of the nanocages to be effectively manipulated. We propose a plausible mechanism for the observed differing structural evolution of the nanocages under different GRR conditions, which is supported by evidence from further experiments.

## Graphical abstract

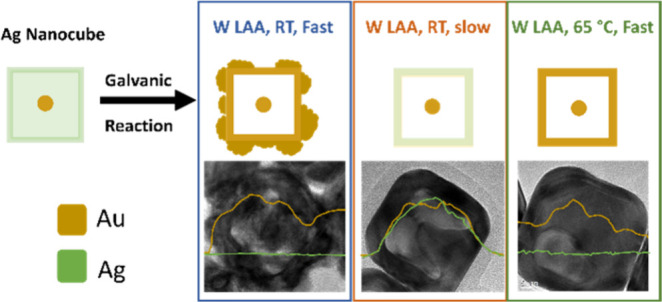

## Introduction

Noble metal nanocrystals, particularly silver (Ag) and gold (Au), have been a focus of interest for many years because of their fascinating optical property, known as localized surface plasmonic resonance (LSPR), which is generated from the collective oscillation of free electrons on the surface of nanoparticles.^1–3^ Such unique properties allow them to be used in widespread applications related to surface-enhanced Raman scattering (SERS), chemical/biological sensing, catalysis, and photonics.^2,4–10^ Among nanocrystals, hollow nanostructures are a distinguished class of plasmonic materials due to a phenomenon where the interaction between plasmons on the inner and outer surfaces of the hollow structure leads to enhanced plasmonic properties, compared to their solid counterparts.^11^ Combining these properties, Au/Ag or Au-based hollow cubic nanostructures are particularly interesting because the sharp corners and edges from cubic geometry contribute to a strong electric field as well as facilitate the tunability of plasmon bands. They have been used in various applications including contrast enhancement agents for tomography,^12^ effective photothermal transducers,^13^ SERS,^14^ colorimetric sensing,^15^ etc. However, the performance of these noble metal hollow cubic nanostructures in applications would be enhanced by more effectively manipulating the morphology and composition during the fabrication process. Better control during the fabrication process would allow for the fine-tuning of their properties.

To fabricate hollow cubic nanostructures with Au or Au/Ag, the most popular strategy is using the galvanic replacement reaction (GRR) as a general route to transform silver nanocubes (AgNCs) used as a template into hollow cubic structures, called nanocages.^16–18^ GRR is an electrochemical process where one metal is oxidized by the ions of another metal with a higher reduction potential.^19^ The reduction potential of Au ions to Au is more positive than that of the Ag ion to Ag. Thus, AgNCs can be oxidized by the Au salt precursor, and Au can be produced through the reaction. The produced Au is confined to the AgNC surface, growing on it and adopting its morphology as the interior Ag is oxidized to produce a hollow structure. Several groups have demonstrated the formation of Au or Au/Ag nanocages by simply titrating HAuCl_4_ into AgNC aqueous solution with or without an additional reducing agent.^15,20^ Recently, several methods have been utilized together with GRR to further control the morphology of these nanostructures. For example, etchant chemicals such as H_2_O_2_ or Fe­(NO_3_)_3_ were introduced to remove Ag from the GRR resulted nanostructure to form cubic nanoframes with controlled porosity and wall thickness.^21,22^ By applying a sequential process of template regeneration and GRR, Ag–Au nanocages with controlled wall thickness can be fabricated and the role of the wall thickness in determining the plasmonic properties has been studied.^21^ However, there has yet to be a systematic study of how the GRR can be manipulated by multiple other factors beyond a reducing agent and how these factors can control the morphology and composition (mono- or bimetallic) of the resulting hollow cubic structures.

Furthermore, nearly all AgNCs used for this structural transformation via GRR were synthesized using a polyol process, which is the most widely used and most successful approach.^23^ The polyol process uses ethylene glycol (EG) or diethylene glycol (DEG) as a solvent and a poly­(vinylpyrrolidone) (PVP) polymer as a capping agent that can also act as a mild reducing agent at 120–180 °C. Although this approach is successful at producing AgNCs, it requires the use of an organic solvent and a high reaction temperature, which is both environmentally unfriendly and economically unattractive. In addition, multiple washings are needed to remove PVP due to its strong adherence to nanoparticles, which increases the difficulty of further modifying the surface of nanoparticles with other ligands. To avoid all these drawbacks, the Xia group lately developed a method using Au nanospheres as a seed to grow AgNCs at mild temperatures.^24^ Instead of PVP, cetyltrimethylammonium chloride (CTAC) can serve as a capping agent for the Ag(100) facet formation. In their work, they demonstrated the size of AgNCs can be varied by changing the gold seed volume, which impacts their corresponding optical properties. They also studied the influence of different ligands (CTAC and cetyltrimethylammonium bromide (CTAB)) on the AgNC formation. Finally, they demonstrate that such AgNCs can be converted into a cubic hollow structure by simply titrating a Au salt precursor. While their work is a great starting point, there are still issues needed to be addressed.

In our work, we first used the Au seed-mediated method to synthesize AgNC with CTAC ligands with slight modification and studied the impact of the concentration of both Au seeds and CTAC surfactant on the morphology and yield of produced AgNCs. Then, we show that compared to conventional GRR, using GRR with a reducing agent, a controlled chemical addition/injection rate, and a controlled reaction temperature results in a dramatically different structural evolution for the hollow structures, also known as a nanocage. These factors impact the deposition rate and diffusion rate of atoms, which in turn affect the final morphology, composition, and optical properties of nanocages. A mechanism for the formation of the resulting nanostructure is proposed and supported with evidence from further experiments.

## Experimental Section

### Chemical and Materials

Tetrachloroauric acid trihydrate (HAuCl_4_·3H_2_O, ≥99.9%), cetyltrimethylammonium bromide (CTAB), cetyltrimethylammonium chloride (CTAC), l-ascorbic acid (LAA), sodium borohydride (NaBH_4_), and silver nitrate (AgNO_3_) were purchased from Sigma-Aldrich. Nanopure water with a resistivity of 18 MΩ cm was used in all experiments. All chemicals were used as received without further purification.

### Preparation of Au Seed Solution

CTAC-capped Au nanospheres were synthesized by using a seed-mediated method according to a previous report with slight modifications.^24^ To prepare the seed, 5.0 mL of 100 mM CTAB was added to 5 mL of 0.25 mM HAuCl_4_ and 0.6 mL of cold 10 mM NaBH_4_. The solution changed from clear to a tea-brown color, indicating the formation of gold seeds. The seeds were then stored away from light at room temperature (RT) for approximately 3 h. A growth solution was prepped by mixing 6.0 mL of 0.50 mM HAuCl_4_ and 6.0 mL of 200 mM CTAC under stirring at 400 rpm followed by an 4.5 mL of 100 mM LAA. Subsequently, 0.3 mL of the Au seed solution was added to the growth solution. The solution then turned wine red, which indicated the growth of the seeds. Au seeds were centrifuged (14,500 rpm for 30 min) and redispersed in nanopure water for further use.

### Preparation of Silver Nanocubes

In a typical procedure, 0.3 mL of gold seeds and 15 mL of 20 mM CTAC solution were mixed, and the mixture was heated at 60 °C under stirring at 400 rpm. After 30 min of heating, 1 mL of 100 mM LAA was added to the solution by hand and then 2.4 mL of 2 mM silver nitrate (AgNO_3_) was injected at a rate of 1 mL/min via a syringe pump. During the injection, the solution began to turn into a cloudy yellow mixture and then an opalescent orange/pink color. After the addition of silver nitrate, the solution was continuously heated and stirred for 4 h. After 4 h, the solution was removed from heat and cooled to RT. The resulting AgNC solution was centrifuged (11,000 rpm for 15 min) and redispersed in nanopure water for further use. Different volumes (0.1, 0.5, 1.3, 1.5, 1.7, and 2.0 mL) of seed solution and various CTAC concentrations (20, 50, and 80 mM) were used to study the impact on AgNC formation.

### GRR between AgNCs and HAuCl_4_ in Various Conditions

To perform conventional GRR, 1 mL of purified AgNCs was mixed with 0.5 mL of nanopure water and 0.5 mL of a 40 mM CTAC solution which was under stirring at 400 rpm. Then, the 0.125 mM HAuCl_4_ solution was added manually into the mixed solution; various volumes were used here, and the specific volumes of additional HAuCl_4_ employed are described in the Results and Discussion section. The solution was stirred for 30 min after the addition.

To perform GRR under LAA, RT, and fast or slow conditions, 1 mL of purified AgNCs was mixed with 0.5 mL of nanopure water and 0.5 mL of a 40 mM CTAC solution which was under stirring at 400 rpm. For the fast condition, the 0.125 mM HAuCl_4_ solution was added manually into the mixed solution using various volumes as described in the Results and Discussion section. Meanwhile, for slow conditions, the 0.125 mM HAuCl_4_ solution was added at a rate of 2 mL/h via a syringe pump into the mixed solution. The solution was stirred for 30 min after the addition.

To perform GRR under LAA, 65 °C, and fast or slow conditions, 1 mL of purified AgNCs was mixed with 0.5 mL of nanopure water and 0.5 mL of a 40 mM CTAC solution which was under stirring at 400 rpm. The mixed solution was then heated at 65 °C in an oil bath for 5 min. Finally, the 0.125 mM HAuCl_4_ solution was added manually into the mixed solution using various volumes, as described in the Results and Discussion section. Meanwhile, for slow conditions, the 0.125 mM HAuCl_4_ solution was added at a rate of 2 mL/h via a syringe pump into the mixed solution. The solution was stirred for 30 min at 65 °C after the addition. Note, before characterization, all he resulting structures were then purified via centrifugation (11,000 rpm for 15 min) and then redispersed into 1 mL of nanopure water.

### Characterization Techniques

All UV–vis spectra were recorded with an Agilent Cary 3500 UV–vis spectrophotometer. Transmission electron microscopy (TEM) images were collected using a JEOL 7200F field emission SEM operated at 30 kV using the TEM mode. HRTEM images of AgNCs, HRTEM images, and EDX line spectra of the nanostructures fabricated from LAA, RT, and fast addition; LAA, RT, and slow addition; and LAA, 65 °C, and fast addition conditions were obtained using an FEI Tecnai F20 instrument (Thermo Fisher Scientific) operated at 200 kV. STEM images and EDX mapping of the nanostructures fabricated from LAA, 65 °C, and slow addition conditions were obtained using a Talos F200x G2 instrument (Thermo Fisher Scientific) operated at 200 kV. TEM images of the same samples were obtained using a JEOL 2100 operated at 200 kV. XRD patterns were measured in the range of 25–90° with a step size of 0.02° and a scan speed of 10°/min while spinning the samples at 10 rpm, employing characteristic Cu Kα radiation having a wavelength of *k* = 1.540593 Å, with a 40 kV voltage and a 15 mA current (Rigaku Miniflex 6G). The full width at half-maximum (fwhm) from different peaks was used in Scherrer’s equation to determine the average crystallite size of the nanoparticles.

## Results and Discussion

### AgNC Synthesis

AgNCs were synthesized by a seed-mediated method using Au nanospheres as seeds, following a recently reported procedure with slight modification.^24^ To synthesize Au seeds, Au nanocrystals were first synthesized by reducing HAuCl_4_ with a strong reducing agent, NaBH_4_, in the presence of CTAB. The synthesized Au nanocrystals were then further grown into Au seeds by reducing additional HAuCl_4_ with a relatively weaker reducing agent, LAA, in the presence of CTAC. The TEM image of synthesized Au seeds is shown in Figure S1a, which shows their spherical shapes with uniform size (∼9 nm). The UV–vis spectrum, shown in Figure S1b, demonstrates that the Au seeds have a sharp absorption peak at 521 nm and have a bright red solution color (inset in Figure S1b).

The AgNCs were formed by further depositing Ag on the as-prepared Au seeds. During this process, typically, Au seeds were suspended in an aqueous solution at 60 °C and the Ag was reduced from AgNO_3_ by LAA in the presence of CTAC. As can be seen in Figure 1a, the synthesized AgNCs have a cubic shape with a length of 38 ± 3 nm. The image also clearly reveals that each nanocube has a dark spherical core at the center. Besides the AgNCs, there are also noticeable impurities in the resulting products such as wires, spheres, right bipyramids, etc. (shown in Figure S2). Figure 1b shows the UV–vis spectrum taken from the synthesized AgNCs aqueous solution, which exhibits one dominant peak located at 450 nm, related to the silver formation, and a shoulder peak at 385 nm, from multiple plasmon resonance.^16,25,26^ We believe that the final peak located at 512 nm comes from the impurities of the products. Since the incident light cannot penetrate silver shells with a thickness beyond 3 nm, the LSPR of the Au seed (located at 521 nm) gets completely blocked by the formed silver shell (the shell thickness is more than 20 nm), which is consistent with the results from theoretical calculations and other reports.^24,27^

**Figure 1:**
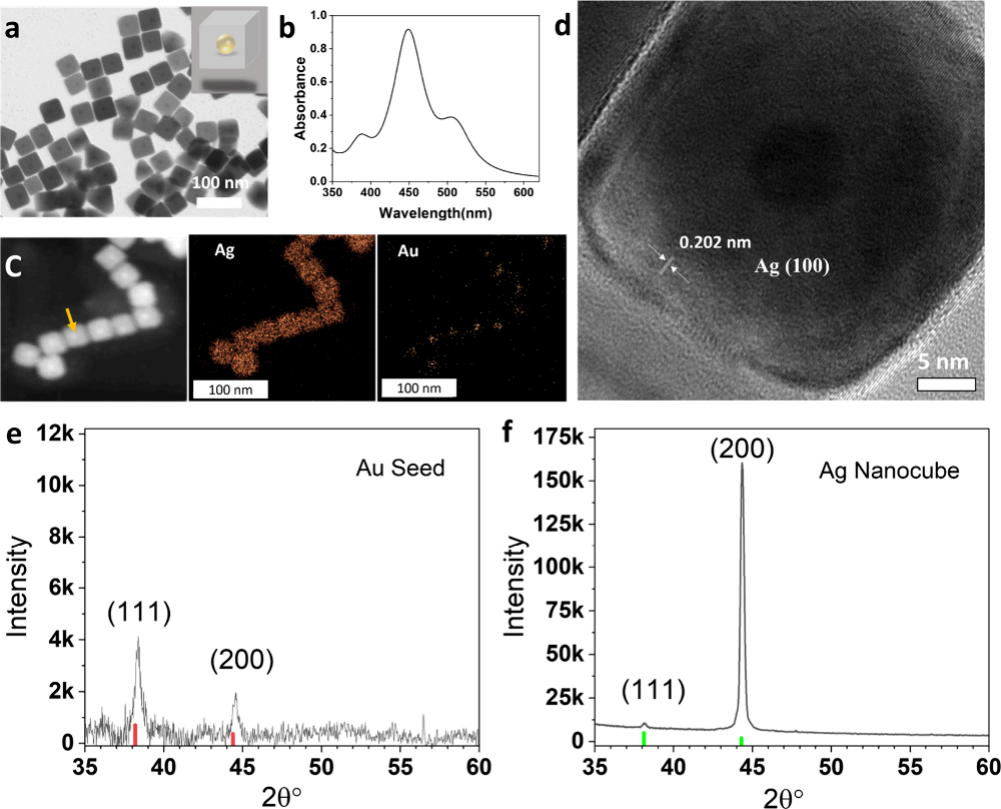
(a) TEM image and (b) UV–vis spectrum of resulting AgNCs. (c) HAADF-STEM image of AgNCs and elemental mapping of Ag and Au. (d) High-resolution TEM image of a single AgNC. X-ray diffraction (XRD) patterns of gold seeds (e) and AgNCs (f).

The resulting AgNCs were further analyzed via high-angle annular dark-field scanning transmission electron microscopy (HAADF-STEM) supported by energy-dispersive X-ray elemental mapping, as shown in Figure 1c. The HAADF-STEM image showed that the AgNC is composed of two components, as indicated by the brighter dot (indicate by the arrow) in the middle of the cube. The elemental mapping of silver and gold shows that the main component is Ag, while the Au seed can be distinguished inside of the nanocube, further confirming that the Au seed is embedded inside the AgNC. An HRTEM image of a single AgNC (Figure 1d) reveals that the average lattice spacing between adjacent fringes was about 0.202 nm, which corresponded to the ⟨100⟩ crystal facets of silver. In addition, the HRTEM image also clearly shows that the gold seed and silver shell are in concentric arrangement, which indicated the symmetric growth of silver on the gold surface. XRD analysis was performed to find the crystalline information on the produced structures, and the results are demonstrated in Figure 1e,f. The XRD pattern of Au seeds (Figure 1e) exhibits formation of a pure gold phase according to the 004-0784 standard card from JCPDS. The obtained spectrum reveals the (111) and (200) peaks, which is in close agreement with previous reports. The XRD pattern of synthesized AgNCs (Figure 1f) also shows the (111) and (200) peaks. However, the intensity ratio of (200)/(111) largely increased and the (200) peak became the most intense peak in the AgNCs reflecting the increment of the (100) facets on the cube surface, which was in agreement with the HRTEM result and previous reports.

### Impact of the Au Seed Volume on AgNC Formation

By adjustment of the volume of Au seed solution added to the growth solution, the Ag shell thickness on the surface of Au seeds can be varied. Figure 2a–f reveals the uniform size and shape of synthesized AgNCs with varying edge lengths obtained by varying the amount of Au seed solutions to the Ag growth solution. With increasing volumes of Au seed solution, the edge length of the resulting AgNCs continuously decreased from 38 to 21 nm (Figure 2g). Manipulating the volume of Au seed in the solution enables effective control over the dimension of the AgNCs in a more straightforward manner compared to the seedless polyol synthesis approach, which requires careful monitoring and quenching of the growth reaction to obtain AgNCs of desired dimensions.^25^

**Figure 2:**
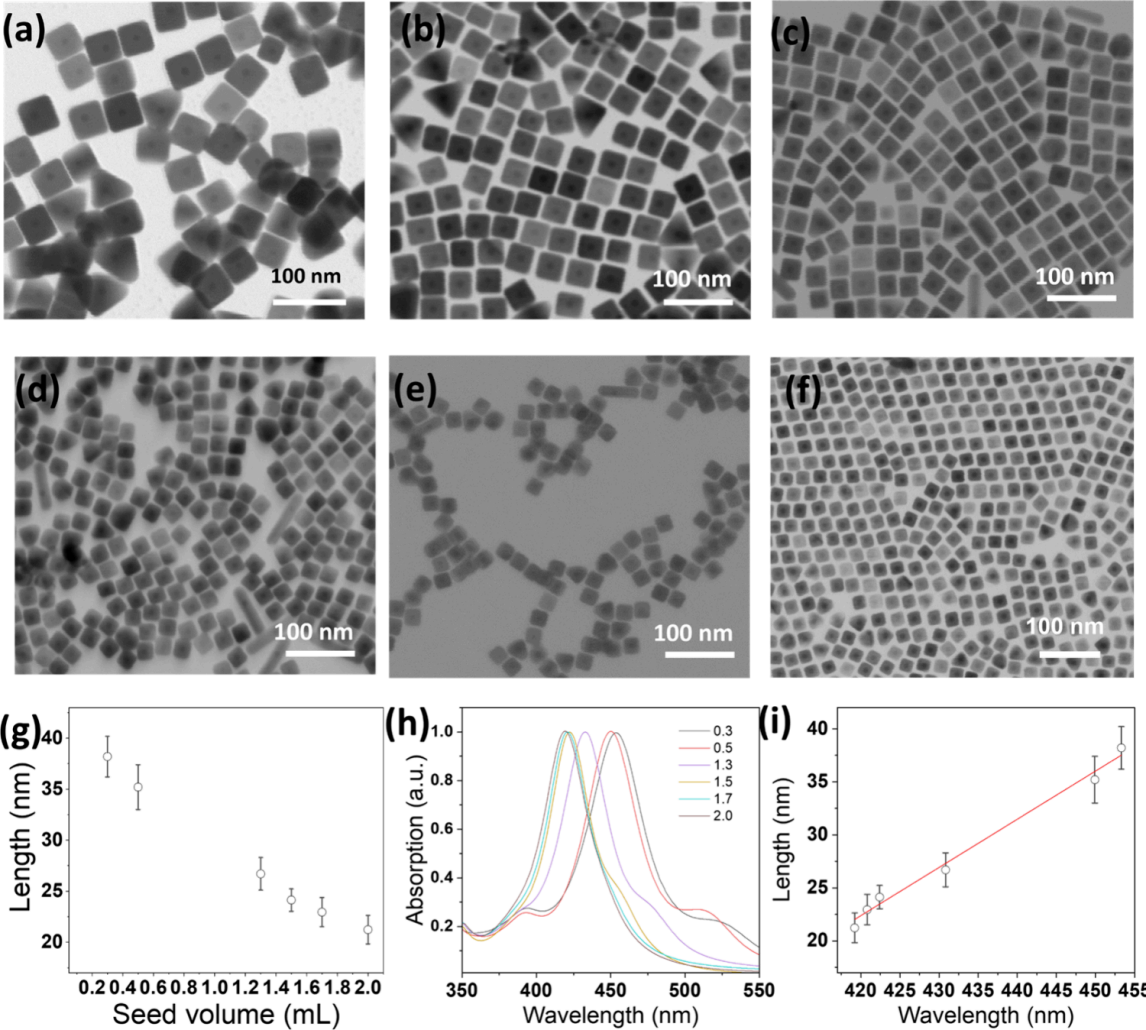
(a–f) Representative TEM images of AgNCs prepared under the same conditions except with different seed volumes: (a) 0.3 mL (default), (b) 0.5 mL, (c) 1.3 mL, (d) 1.5 mL, (e) 1.7 mL, and (f) 2.0 mL. (g) Plot summarizing the dependence between the length of the resulting AgNC and the gold seed volume. (h) UV–vis spectrum of resulting AgNCs shown in (a–f). (i) Plot showing the dependence between the length of the resulting AgNC and the major LSPR peak position.

The corresponding extinction spectra of the resulting AgNCs reveal a progressive blueshift as the dominant LSPR peak moves from 453 to 419 nm as the AgNC is smaller (Figure 2h). In addition, the shoulder peak from multiple plasmon resonance, initially around 385 nm, was diminished with higher Au seed volumes. Furthermore, the increase in the LSPR wavelength along with the edge length of the nanocubes was found to be linear over the size range investigated here, which is in agreement with previous reports (Figure 2i).

When the Au seed solutions are reduced to 0.1 mL from the default volume (0.3 mL), the resulting nanomaterials show the formation of AgNCs with larger length (∼45 nm) as well as significant amounts of nanorods/nanowires shown in the products (Figure S3). In this case, the ratio of silver ions to individual gold seeds is much higher; the cube shape is less well-maintained. Instead, the silver will keep depositing on the side of the cube shape and forming the elongated wire and nanorod morphologies.

### Impact of the CTAC Surfactant on AgNC Formation

We believe that one way to prevent the formation of elongated nanowires and nanorods, in the case when the Au seed solution has a relatively low volume, is to add sufficient CTAC surfactant. Cl^–^ from CTAC serves as a capping agent for the Ag(100) facets.^16^ Since the tail of CTAC is short and thus has less steric interaction at the corner and edges of the cube, silver ions transport and form sharper cubes. In the case of 0.1 mL of Au seed, the ratio of silver ions to individual gold seeds is much higher. As the particle grows, the CTAC packing density around the particle will decrease, and no additional CTAC can fill up the packing density to maintain the cubic morphology. Thus, the silver ions can continue to deposit on the particle and form elongated nanorods and wires instead of forming sharp corners for nanocubes. To verify our assumption, CTAC surfactant concentration was varied from a baseline of 20 mM to 50 and 80 mM while the seed volume and other experimental conditions were kept the same. The results are shown in Figure 3a–c, which clearly shows that with increased [CTAC], the nanowires mostly disappeared and nanorods were significantly reduced. The size of the nanocube formed under increased [CTAC] is slightly increased as shown in Figure S4. In addition, there is a significant amount of polydispersed spheres formed in higher [CTAC], which we believe are formed due to silver self-nucleation. Besides serving as a relatively strong capping agent toward the (100) facets, with additional CTAC, the Cl^–^ from CTAC will also form AgCl nanoparticles, and under the condition of the LAA reducing agent at 60 °C, they would change to element Ag and then Ag_*n*_ nuclei would be generated, followed by their evolution into single-crystal seeds, as reported previously.^28^

**Figure 3:**
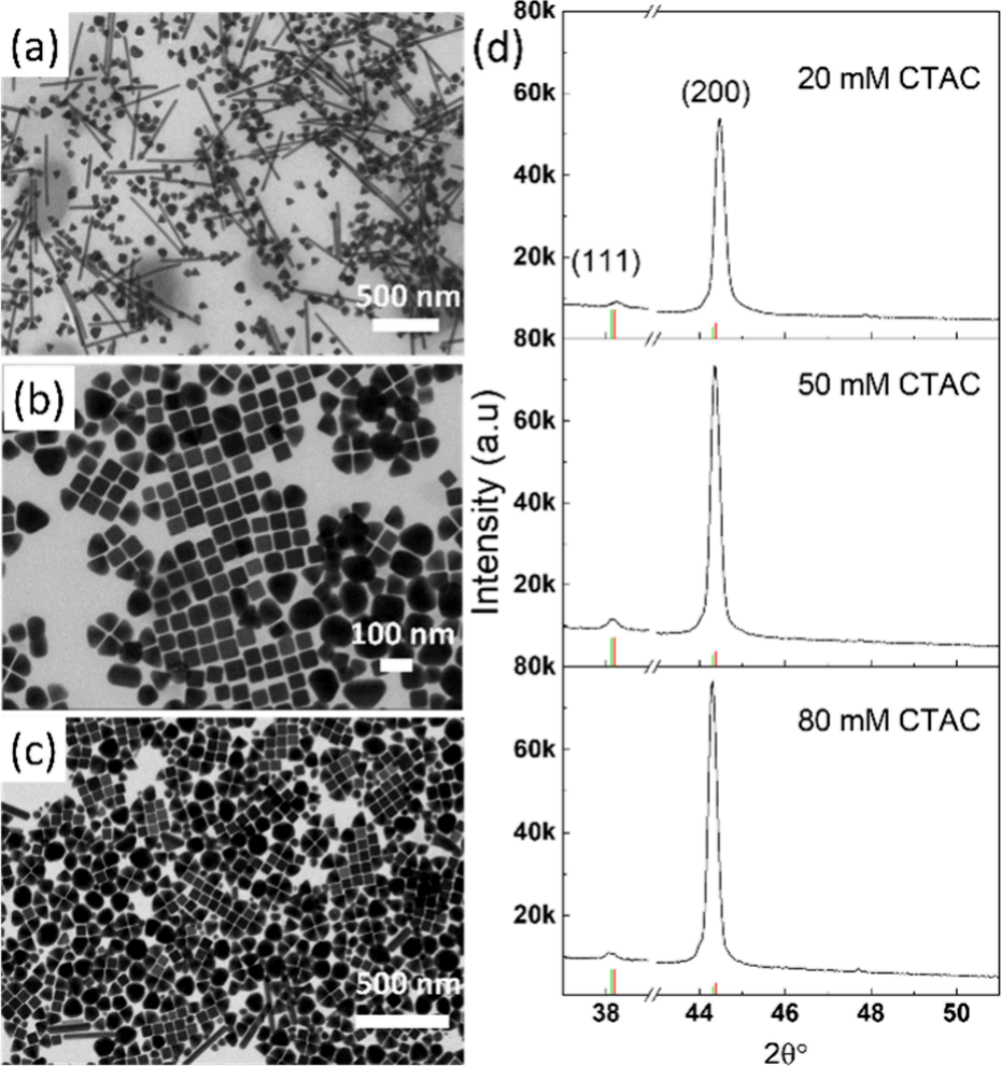
(a–c) STEM images of AgNCs synthesized under CTAC concentrations of 20 mM (a), 50 mM (b), and 80 mM (c). (d) XRD patterns of AgNCs shown in (a–c). The green vertical bars are corresponding to the JCPDS 04-0783 standard card for the metallic silver phase. The red vertical bars are corresponding to the JCPDS 004-0784 standard card for the metallic gold phase.

XRD analysis was performed to find the crystalline information on produced structures under [CTAC] at 20, 50, and 80 mM, and the plots as well as the data analysis results are demonstrated in Figure 3d and Table S1. The peaks are assigned to the diffraction of the (111) and (200) planes of face-centered cube (fcc) silver according to the 04-0783 standard card of the metallic silver phase from JCPDS, whose peak appeared at 2θ = 38.1° for the (111) and 44.4° for the (200), indicated as green vertical bars in Figure 3d. The average size of the NCs can be calculated using the Scherrer equation based on the full width at half-maximum (fwhm) of the (200) diffraction peak, which is about 35–38 nm for all conditions. The (200) peaks in all the XRD patterns are much stronger than the commonly strongest (111) peak, which indicates the successful formation of the cubic shape with six exposed facets as the (100) planes while the intensity ratio of the (200) to (111) is much stronger for the sample under 80 mM CTAC, which is understandable since the CTAC facilitates the (200) plane formation. The peak location is interesting to compare among all samples. For the lowest [CTAC], the (200) and (111) peaks are at 44.47 and 38.23°, respectively, which drifted away from the silver metallic reference indicated by the green tick and closer to the gold metallic reference, indicated by the red tick. We believe that these changes are due to the greater impact of gold seeds in the sample under these synthesis conditions. Gold seeds are incorporated into the wire and nanorods as well as the nanocubes. The gold core in the wire and nanorods is less deeply embedded in the silver material, as compared to the nanocubes, so it will have a larger contribution to the XRD analysis when using the lowest [CTAC]. With increased [CTAC], the peaks of (200) and (111) are all shifted to lower 2θ, which are closer to the reference metallic silver phase, as shown in Figure 3d (80 mM CTAC XRD pattern). This is because with increasing CTAC, the nanowire mostly disappeared and nanorods are significantly reduced, while silver polyspheres are formed. Thus, the relative impact of the gold seed should be reduced, which is in agreement with the XRD results. These results indicate that having a balanced ratio of silver ion to gold seed, as well as using an appropriate concentration of the CTAC surfactant, is crucial to form high-yield AgNCs.

### Controlled Structural Change of AgNCs to Au Hollow Cubic Nanostructures (Nanocages) under GRR

GRR is a popular approach to transform solid Ag shells into hollow Au shells. Since the reduction potential of Ag is lower than Au (Ag^+^/Ag: + 0.8 V, Au^3+^/Au: + 1.5 V), GRR will spontaneously take place by introducing Au precursors such as HAuCl_4_. While GRR is an effective approach for transforming Ag shells into hollow Au shells, this can often result in either a thin frame or decomposed fragments that are not ideal for further manipulation. To study and control the silver shell structural evolution under GRR and thus the resulting properties of the hollow Au shell, we will introduce several factors including using the reducing agent LAA, slowing the injection rate, and increasing the temperature beyond RT to the GRR process and study their effects on the resulting morphology.

Before undertaking these steps, we first investigate the GRR-induced optical property and structural evolution of AgNCs into hollow Au shells (nanocages) without changing these factors, which we refer to as conventional GRR (no LAA, RT, and standard injection rate). Figure 4a shows the UV–vis spectra and solution color change of the resulting nanostructures after manually titrating different amounts of HAuCl_4_ solution into the large-sized AgNC solution created using CTAC, as discussed earlier in this paper (38 ± 3 nm). The initial AgNCs display the peak at around 450 nm, and with additional HAuCl_4_, the peak progressively redshifts and a weak shoulder peak appears around 550 nm, indicating the formation of Au.^12,29^ In addition, the intensity of spectra is decreased, which indicates a loss of silver content (from 50 to 300 μL). When the amount of HAuCl_4_ was more than 500 μL, the intensity of the spectra was very low, and no clear peak was resolved, indicating very low metal content in solution. TEM images show the representative morphologies of the resulting nanostructures. After introducing 0.05 mL of HAuCl_4_ (Figure 4b), the surface of the particles appears to have craters when Au is deposited on the outer Ag surface, as expected in GRR. This agrees with the optical spectrum observation, where the peak is redshifted and has a lower intensity due to the loss of Ag and formation of Au. Such craters were expanded to holes with addition of HAuCl_4_ (300 μL, 0.5 mM, seen in Figure 4c), and upon further addition of HAuCl_4_ (500 μL, 0.5 mM), the cube was completely hollowed out and resulted in the nanostructure of a Au core inside a Au nanobox (Figure 4d). Note that the core of the AgNC is gold, which will not be oxidized in the GRR process. The corresponding UV spectrum in Figure 4a shows that the spectrum for the 500 μL addition (dark goldenrod color) has no significant peak and the overall intensity is very low, which is reasonable since the formed nanobox has very thin walls. It is important to know that with the released electron from silver oxidation, the AuCl_4_^–^ ions are reduced to Au atoms simultaneously on the outer surface and interdiffusion between Au and Ag atoms causes the walls to form an alloy. With further addition of HAuCl_4_ (1 mL), the nanobox becomes fragments (Figure 4e) and the solution shows a pink color, indicating gold. This is because the walls are dealloyed as the HAuCl_4_ reacts with the Ag atoms selectively, generating craters that coalesce into holes in the walls of the nanobox, which could collapse depending on the rate of the GRR. These results obtained using our AgNCs through the conventional GRR process agree with previously published work using cubic or other shaped silver nanostructures created in more traditional ways.^30–32^

**Figure 4:**
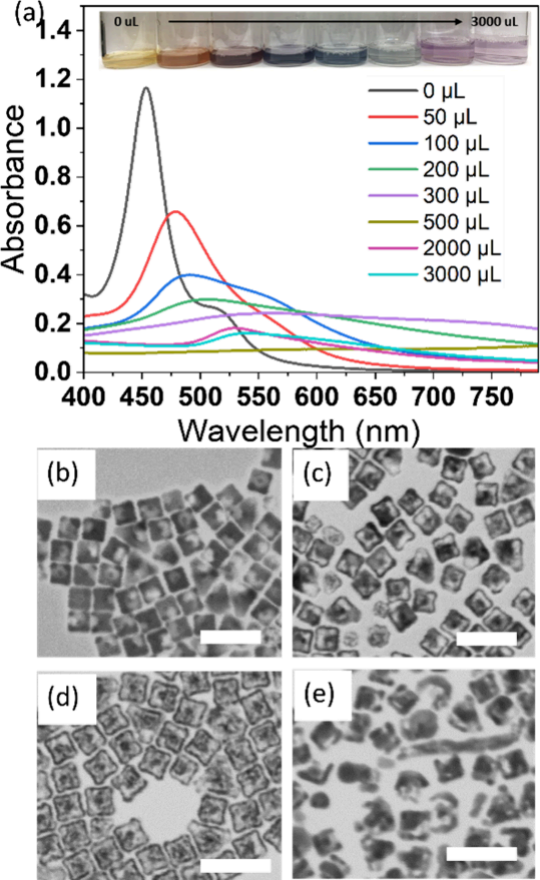
(a) UV spectra of resulting solution prepared by titrating different volumes of a HAuCl_4_ solution into the AgNC solution; inset: the corresponding solution color with varying amounts of HAuCl_4_ addition. (b–e) STEM images of resulting nanostructures after titration of HAuCl_4_ solution with (b) 50 μL, (c) 300 μL, (d) 500 μL, and (e) 1000 μL. Scale bar: 100 nm.

We now vary three conditions for the GRR to study the impacts on the shape and morphology of the resulting Au hollow nanostructure. The baseline is what was performed previously in this section: performed at conditions without the reducing agent LAA, at RT, and manually titrating HAuCl_4_ solution relatively quickly. We then change each of these three conditions: carrying out at conditions with LAA, titrating the HAuCl_4_ solution relatively slowly at a 2 mL/h rate, and performing the GRR at 65 °C. All three conditions here include the LAA; the first has no other changes from the baseline, the second uses higher temperatures with baseline titration speed, and the third is at RT with slow titration speed. Detailed experimental procedures can be found in the Experimental Section.

Figure 5 shows the optical properties of the resulting solution prepared through GRR of AgNCs with different amounts of HAuCl_4_ under the indicated conditions. Compared to the conventional GRR, in general, under all three conditions, the peak remained very robust across the spectral range regardless of the amount of gold titration, while the location of the peak shifted across a broad spectral range (Figure 5a–c). Specifically, as the amount of HAuCl_4_ increased, the wavelength and intensity of the plasmon resonance peaks have a less dramatic redshift and decrease, respectively. When the volume of HAuCl_4_ solution reaches 300 μL (purple lines in Figure 5a–c), the gold shoulder peaks become visible for all three conditions and the peak intensity progressively increases as the volume of HAuCl_4_ solution continues to increase. The results indicate that the GRR path is still happening, but the degree is much lower than the conventional GRR due to the introduction of LAA. Introducing the reducing agent LAA generates a parallel reduction path that can compete with and thus suppress the GRR. This has been observed by our previous work regarding suppressing GRR between Ag and Pt^2+^ as well as other related works.^4,33^

**Figure 5:**
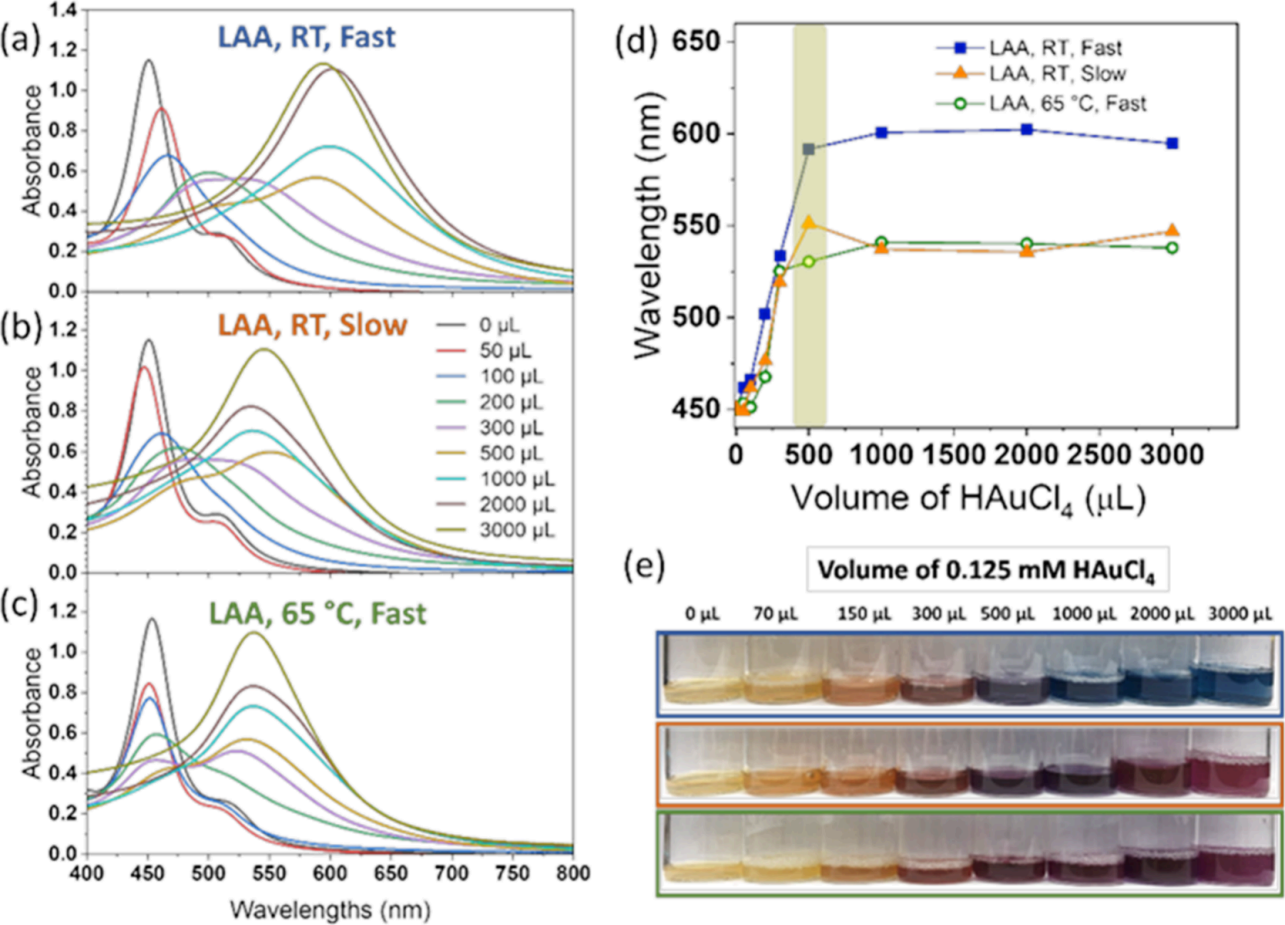
(a–c) UV–vis spectra of resulting solution prepared through GRR of AgNCs with HAuCl_4_ under various conditions: (a) LAA, RT, and fast addition; (b) LAA, RT, and slow addition; (c) LAA, 65 °C, and fast addition. (d) Extinction peak wavelength as a function of the volume of HAuCl_4_ solution. (e) Picture of the solution color after adding various volumes of HAuCl_4_ solution under three conditions including LAA, RT, and fast addition (blue box); LAA, RT, and slow addition (orange box); and LAA, 65 °C, and fast addition (green box).

Figure 5d summarizes the extinction peak wavelength change as a function of the volume of HAuCl_4_ solution under the three conditions, noting that when there were two peaks in spectra, the peak with a longer wavelength was recorded. When adding less than 500 μL of HAuCl_4_, the resulting extinction peaks for all three conditions have a gradually similar redshift from ∼450 to ∼525 nm, while at 500 μL of HAuCl_4_, the resulting peak wavelength from the LAA/RT/fast addition condition has a much bigger shift to a 590 nm wavelength. When adding more than 500 μL of HAuCl_4_, the peak shifted mildly for all conditions, and the resulting peak wavelengths are similar between the LAA/RT/slow addition condition and the LAA/65 °C/fast addition condition, about 540 ± 10 nm. Such optical properties can also be observed by the color of the resulting solutions shown in Figure 5e. When the addition of HAuCl_4_ amount is less than 500 μL, the solution color for all three conditions is similar, indicating that the changes of AgNC are similar in all cases. While using 500 μL of HAuCl_4_ addition, the resulting solution color for the LAA/RT/fast addition condition shows a bluish color, the resulting solution colors from the other two conditions show a purple color, indicating significant differences of the resulting nanostructures. When more than 500 μL of HAuCl_4_ is added, the resulting solution colors show no significant change.

TEM was used to check the morphological changes of the resulting nanostructures prepared through GRR of AgNCs with HAuCl_4_ under the three varying conditions, and Figure 6 shows the representative TEM images of the resulting nanostructures after being reacted with various volumes of HAuCl_4_ under the three conditions. With 50 μL of addition under all three conditions, the resulting NC does not show much etching, and we are unable to observe the small pinholes seen under conventional GRR, a dramatically different result (shown in Figure 4b). The edge lengths of the NC for all three conditions were measured as 36.8 ± 1.9, 36.6 ± 1.5, and 36.2 ± 1.9 nm, essentially the same as each other and of the original AgNC. When the volume of HAuCl_4_ was increased to 500 μL, it is interesting to notice that NCs resulting from the LAA/RT/fast addition condition mostly exhibit hollow cubic nanocages, while NCs resulting from both the LAA/RT/slow addition condition and LAA/65 °C/fast condition mostly exhibit large holes instead of a completely hollow structure. The edge length of the NC from LAA/RT/fast addition condition was increased from 36.8 ± 1.9 to 46.7 ± 1.9 nm, a little larger change than the NC from LAA/RT/slow addition, which increased from 36.6 ± 1.5 to 43.4 ± 2.2 nm, as well as the NC from LAA/65 °C/fast addition, which increased from 36.2 ± 1.9 to 42.9 ± 1.5 nm. When the volumes of HAuCl_4_ increase further to 1000 and 3000 μL, under LAA/RT/fast addition conditions, the resulting NCs become completely hollow cubic nanocages and with more gold atoms generated from the reaction, the formed gold shell has both a thicker and rougher coating (Figure 6c,d); the size for 1000 μL is 50.3 ± 2.4 nm and for 3000 μL is 62.3 ± 3.0 nm. Meanwhile, under both LAA/RT/slow addition and LAA/65 °C/fast addition conditions, the resulting NCs maintain a cubic nanocage with smooth edge surfaces and the shell becomes thicker when the gold deposits more (Figures 6g,h and 6k,l). With 1000 μL of HAuCl_4_, the edge lengths under LAA/RT/slow addition and LAA/65 °C/fast addition conditions are 45.9 ± 2.0 and 44.8 ± 2.5 nm, respectively. With 3000 μL of HAuCl_4_, the edge lengths, under LAA/*RT*/slow addition and LAA/65 °C/fast addition conditions, are 50.5 ± 1.5 and 50.4 ± 3.2 nm, respectively. These are essentially identical lengths under both conditions. This finding is not surprising given the similar UV shift as well as the similar solution color with these two conditions. We believe that in the absence of LAA, changes in the titration rate and reaction temperature alone are insufficient to alter the outcome. Without LAA to serve as a competing reaction, the nanostructure is expected to follow the conventional GRR pathway, initially forming a hollow structure and eventually collapsing, depending on the GRR rate.

**Figure 6:**
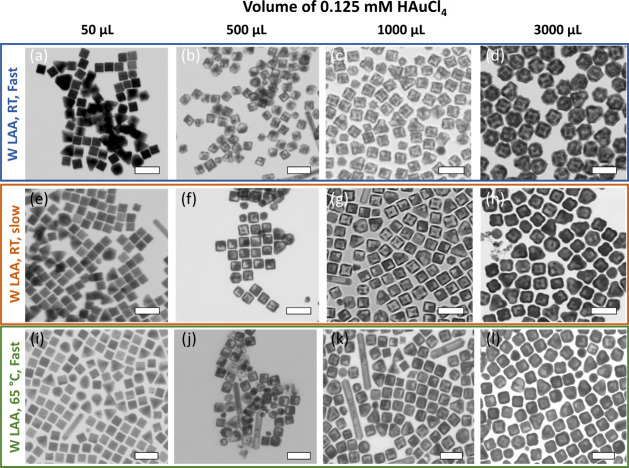
TEM images of resulting nanostructures prepared through GRR of AgNCs with HAuCl_4_ under various conditions: (a–d) results of using reducing agent LAA, at RT, and adding HAuCl_4_ manually (fast) with various volumes including 50 μL (a), 500 μL (b), 1000 μL (c), and 3000 μL (d). (e–h) Results of using LAA, RT, and adding HAuCl_4_ at a slow rate (2 mL/h) with various volumes including 50 μL (e), 500 μL (f), 1000 μL (g), and 3000 μL (h). (i–l) Results of using LAA, high temperature (65 °C), and adding HAuCl_4_ manually (fast) with various volumes including 50 μL (i), 500 μL (j), 1000 μL (k), and 3000 μL (l). Scale bar: 100 nm.

To better understand the structure and composition information under the three conditions, we used high-resolution TEM (HRTEM) to obtain the morphology as well as energy-dispersive spectroscopy (EDS) to correlate the structures of individual nanoparticles with their compositions for the resulting structure when using 3000 μL of HAuCl_4_. Note that, in the EDS results, the green line with blue end circle indicates the line scan for EDS and their result profile is on top of the image. Due to the software limitation, it is impossible to separate the line and particle for clearer display. However, the elemental profiles are separately plotted in the Supporting Information. Under the LAA/RT/fast addition conditions, the resulting NCs clearly reveal that the structures are hollow with a rough surface, as shown in Figure 7a and Figure S5a. The EDS result shown in Figure 7d and the enlarged line profile in Figure S5d show that the resulting structure contained Au while no Ag was detected. In the conditions of LAA/RT/slow addition and LAA/65 °C/fast addition, the resulting NCs reveal that the structures are hollow with smooth edges and corners, as shown in Figure 7b,c and Figure S5b,c. These observations are consistent with TEM in Figure 6h,i. The EDX results show interesting differences. Under the condition of LAA/RT/slow addition, the resulting NC contains both Au and Ag, as shown in Figure 7e and the enlarged line profile in Figure S5e, while under the LAA/65 °C/fast addition condition, the resulting NC contains Au while Ag is not able to be detected, as shown in Figure 7f and the enlarged line profile in Figure S5f.

**Figure 7:**
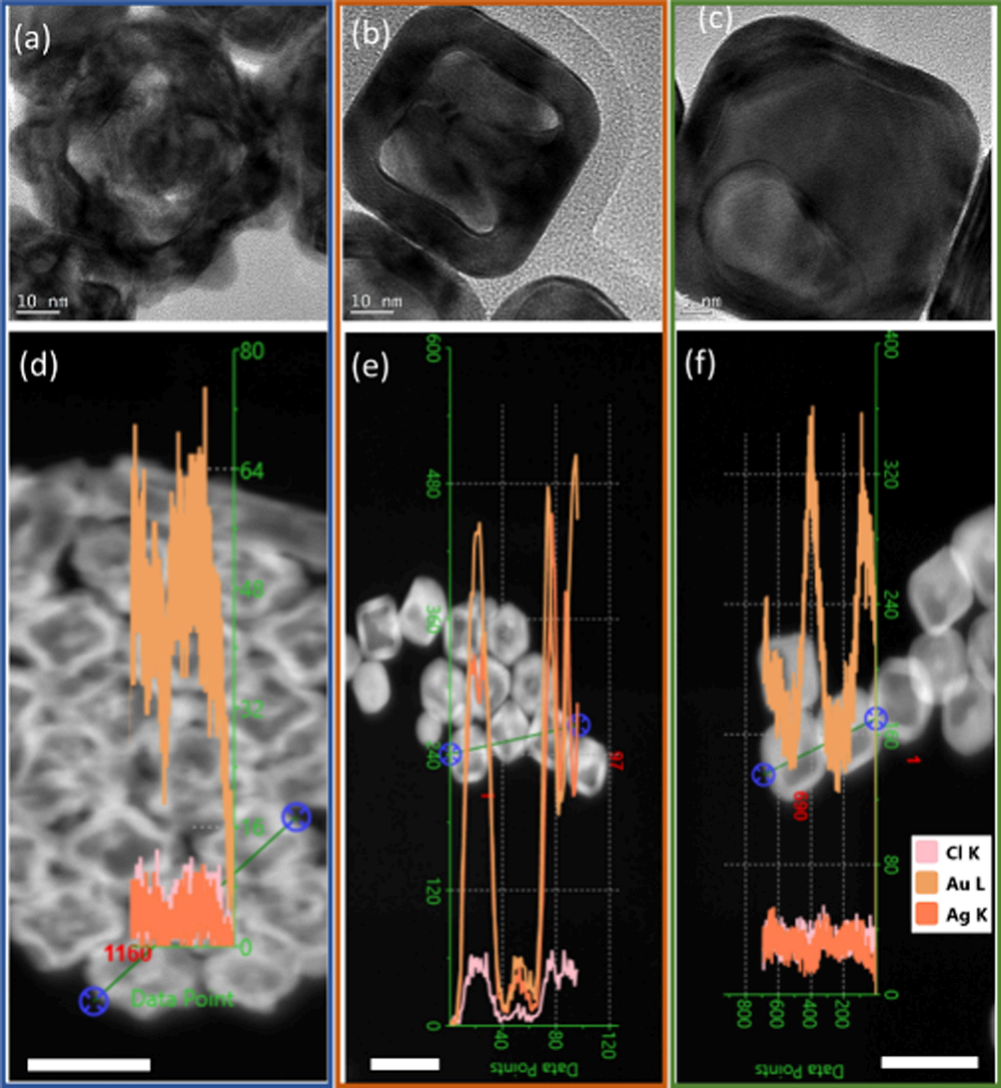
(a–c) TEM images of individual nanostructures with 3000 μL of HAuCl_4_ obtained under various conditions: (a) LAA/RT/fast addition, (b) LAA/RT/slow addition, and (c) LAA/65 °C/fast addition. (d–f) Line-scan EDX spectra and HAADF-STEM images of elemental Ag, Au, and Cl that were recorded along the green line on top of the nanoparticles from (a–c) results: (d) LAA/RT/fast addition, (e) LAA/RT/slow addition, and (f) LAA/65 °C/fast addition.

It is known that the morphology structure is determined by the rate of atom deposition (*V*_dep_) and the rate of surface diffusion (*V*_diff_) of the adsorbed atoms (adatoms). The *V*_dep_ is influenced by the concentration of the newly formed atoms in the reaction solution, which can be controlled by the titration rate for HAuCl_4_ solution. The *V*_diff_ can be controlled by the temperature of the reaction and its rate increase with increased temperature since surface diffusion is a thermally promoted process.

Here, we propose a plausible mechanism that can account for the differing morphologies and elemental changes under three conditions. Under LAA/R*T*/fast addition conditions, the titration rate is high, which produces a high concentration of newly formed Au atoms, and the reaction temperature does not promote the surface diffusion. Thus, the ratio of *V*_dep_/*V*_diff_ is large since the surface diffusion value is very small, and the resulting structure shows a rougher surface and larger particles as the atoms quickly deposit on the surface of the nanostructure instead of migrating to other locations. In the case of LAA/RT/slow addition conditions and LAA/65 °C/fast addition conditions, the ratio of *V*_dep_/*V*_diff_ is expected to decrease by slowing the titration rate or increasing the reaction temperature. This promotes surface diffusion, and thus, adatoms can migrate to other locations, such as edges, side faces, and the inside of the nanostructure, leading to formation of nanostructures with smooth surfaces and smaller size. In terms of elemental composition, Ag is still present in the LAA/RT/slow addition condition instead of the two other conditions. This is because the titration rate of HAuCl_4_ solution is slow, so LAA can reduce the newly oxidized Ag^+^ by HAuCl_4_ solution via GRR. This cannot be achieved in the case of fast addition since LAA is mainly reducing large amounts of HAuCl_4_ solution in solution. To validate this proposed mechanism, we conducted another experiment under the LAA/65 °C/slow addition condition. In this case, the *V*_dep_/*V*_diff_ is small, so we expect that the surface will be smooth and both the Au and Ag elements will be present in the final structure. As shown in Figure 8, we indeed obtained nanostructures with smooth surfaces. As indicated by EDX mapping, both Au and Ag are present in the nanostructure. This is related to the results of the Xia group, who have also used the temperature and injection rate to control the ratio of *V*_dep_/*V*_diff_ and thus morphology structure, in their case for Pd nanocrystals.^34^

**Figure 8:**
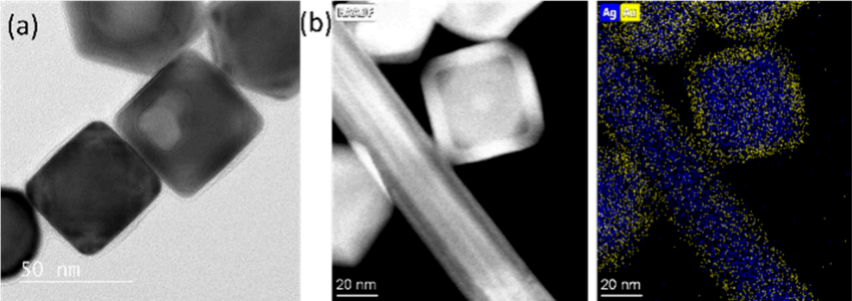
(a) TEM image of nanostructures with 3000 μL of HAuCl_4_ obtained under LAA, 65 °C, and slow addition conditions. (b) HAADF-STEM image and its corresponding EDX mapping.

## Conclusions

In summary, AgNCs with a size of 38 nm were successfully synthesized by using a gold seed-assisted method using an aqueous system. By increasing the amount of the Au seed, the edge length of the AgNCs decreased, and eventually, when the ratio of [Ag^+^] to individual gold seed became too high, the fraction of nanostructures with cubic morphology declined, while significant amounts of nanowires and nanorods appeared. In addition, our CTAC concentration study shows that a moderate amount of CTAC promotes the formation of cubic morphology, though an excessively high concentration can cause silver self-nucleation, increasing the amount of impurity products in synthesis. Furthermore, we studied the structural change of AgNCs created under these conditions under both conventional GRR, as well as modified GRR including the use of a reducing agent, adjusting the injection rate, as well as increasing the reaction temperature. Introducing the reducing agent generates a parallel reduction path, which can compete with and suppresses the GRR. The injection rates and reaction temperature impact the ratio of adatom deposition rate to diffusion rate, thereby controlling the morphology and composition of resulting nanostructures. These results indicate that GRR with appropriate additional factors can provide versatile routes for fine tailoring the morphology and composition of nanocages. This study can provide guidance on rational design of GRR fabricated nanocages with desired surface roughness, interior porosity, and metal composition.

## Supplementary Material



## Notes

TEM images, XRD data analysis, HRTEM, and EDX line spectra of resulting nanostructures (PDF)
