# Supplementary material for: Studying the Synthesis of Silver Nanocubes and Their Structural Evolution under Controlled Galvanic Reactions
Source: J Phys Chem C Nanomater Interfaces. 2025 Jul 25;129(31):14204–13. doi: 10.1021/acs.jpcc.5c03561 (PMC12337138; doi:10.1021/acs.jpcc.5c03561)
Supplement: Supplementary file 1 [file jp5c03561_si_001.pdf]

SUPPORT INFORMATION

Studying the Synthesis of Silver Nanocubes and Their Structural Evolution Under Controlled Galvanic Reactions

Anika Guo[1], Nicolas Hall[1], Teagan Hamlett[1], John R. Crockett[2], Annabella Talbott[1], Tosin Ogunrinola[1], Ayomide Oluwafemi[1], Meghan Burke[1], Qian Chen[2], and Ying Bao[1]*

1. Department of Chemistry, Western Washington University, Bellingham, WA, USA.

2. Department of Material Science and Engineering, The Grainger College of Engineering, University of Illinois Urbana-Champaign, Urbana, IL, USA.

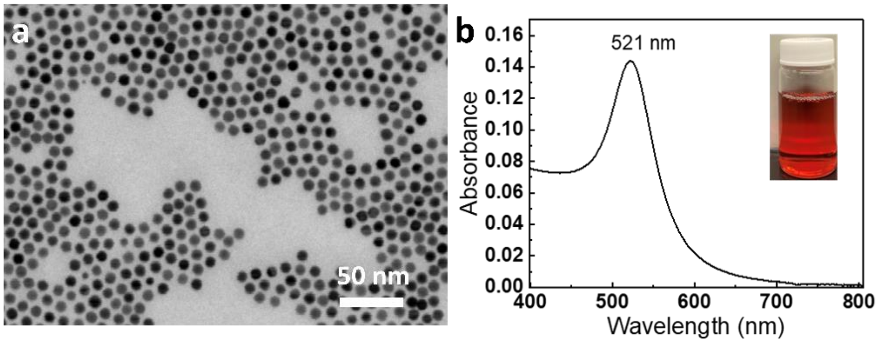

Figure S1. (a) TEM image and (b) UV-Vis spectrum of the Au seeds; inset: photo of the Au seeds solution.

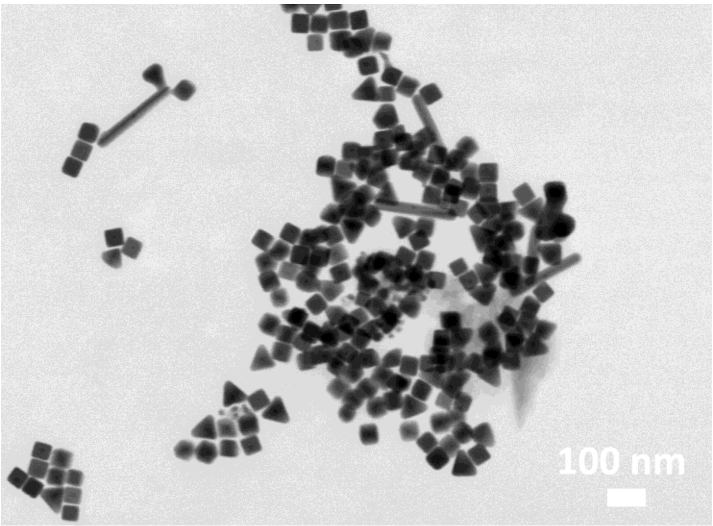

Figure S2. TEM image of synthesized AgNC with byproducts.

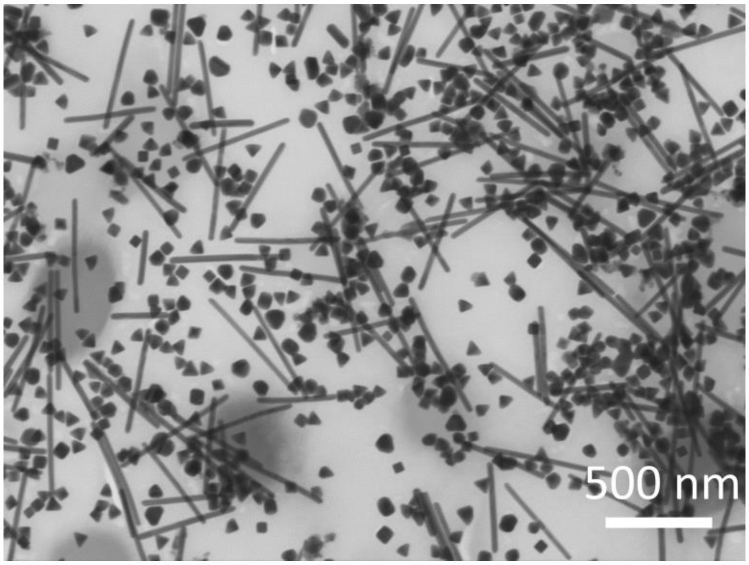

Figure S3. A representative TEM image of AgNCs prepared with 0.1 mL gold seed solution.

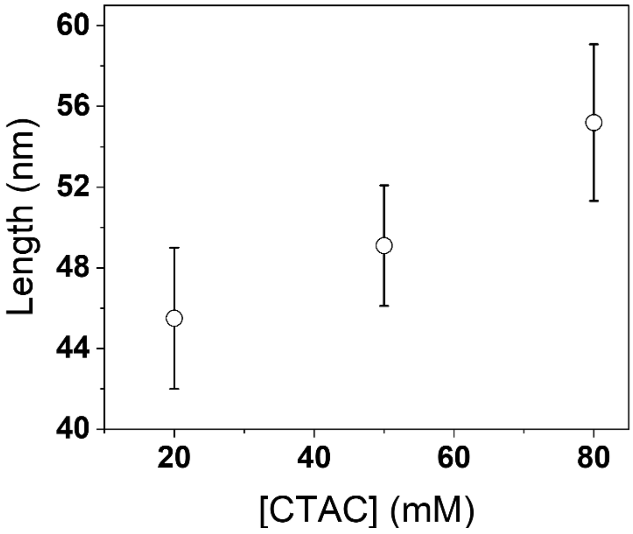

Figure S4. A plot of the resulted AgNC lengths against the concentration of CTAC.

Table S1 XRD information of AgNCs synthesized under various CTAC concentration.

[CTAC]	Peak Intensity of (200)/(111)	Peak Location		(200) Peak		
		(200)	(111)	FWHM	Wavelength	Crystal size
20 mM	5.69	44.47	38.23	0.25	0.15406	35
50 mM	6.10	44.36	38.16	0.23	0.15406	38
80 mM	6.81	44.30	38.10	0.25	0.15406	35

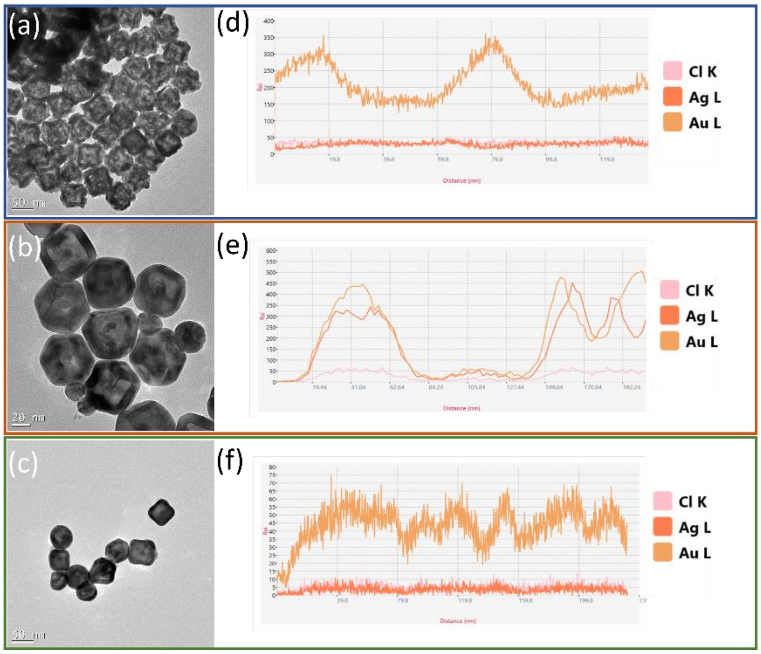

Figure S5. (a-c) TEM images of nanostructures with 3000 µL HAuCl4 obtained under various condition: (a) LAA, RT and fast addition; (b) LAA, RT and slow addition; (c) LAA, 65 °C and fast addition. (d-f) zoom in Line-scan EDX spectra of elemental Ag, Au and Cl from Figure 7d-f.
